# *Premolis semirufa* (Walker, 1856) Envenomation, Disease Affecting Rubber Tappers of the Amazon: Searching for Caterpillar-Bristles Toxic Components

**DOI:** 10.1371/journal.pntd.0001531

**Published:** 2012-02-28

**Authors:** Isadora Maria Villas-Boas, Rute Maria Gonçalves-de-Andrade, Giselle Pidde-Queiroz, Suely Lucia Muro Rais Assaf, Fernanda C. V. Portaro, Osvaldo A. Sant'Anna, Carmen W. van den Berg, Denise V. Tambourgi

**Affiliations:** 1 Immunochemistry Laboratory, Butantan Institute, São Paulo, São Paulo, Brazil; 2 Genetics Laboratory, Butantan Institute, São Paulo, São Paulo, Brazil; 3 Department of Pharmacology, Oncology and Radiology, School of Medicine, Cardiff University, Cardiff, United Kingdom; Hebrew University-Hadassah Medical School, Israel

## Abstract

**Background:**

The caterpillar of the moth *Premolis semirufa* (Lepidoptera: Arctiidae), commonly named *Pararama*, is endemic of the Amazon basin. Accidental contact with these caterpillar bristles causes local symptoms such as intense heat, pain, edema and itching which last for three to seven days; however, after multiples contacts, it may induce joint-space narrowing and bone alteration, as well as degeneration of the articular cartilage and immobilization of the affected joints. Specific treatment for this disease does not exist, but corticosteroids are frequently administered. Despite of the public health hazard of *Premolis semirufa* caterpillar poisoning, little is known about the nature of the toxic components involved in the induction of the pathology.

**Methodology/Principal Findings:**

Here we have investigated the biological and immunochemical characteristics of the caterpillar's bristles components. Analysis of the bristles extract in *in vitro* assays revealed the presence of proteolytic and hyaluronidase activities but no phospholipase A_2_ activity. *In vivo*, it was observed that the bristles extract is not lethal but can induce an intense inflammatory process, characterized by the presence of neutrophils in the paw tissues of injected mice. Furthermore, the bristles components stimulated an intense and specific antibody response but autoantibodies such as anti-DNA or anti-collagen type II were not detected.

**Conclusion:**

The results suggest that *Premolis semirufa* caterpillar bristles secretion contains a mixture of different enzymes that may act together in the generation and development of the clinical manifestations of the Pararama envenomation. Moreover, the high immunogenicity of the caterpillar bristles components, as shown by the generation of high antibody titers, may also contribute to the induction and establishment of the inflammatory disease.

## Author Summary

Pararama, the popular name of the larval form of the moth *Premolis semirufa* inhabits rubber plantations in the Amazon region and the accidental contact of the skin with the caterpillar's bristles or cocoons results in immediate and intense heat, pain, edema, and itching. In many cases a chronic inflammatory reaction with immobilization of the joints occurs. The current study has evaluated the biological and immunochemical characteristics of the Pararama caterpillar bristles extract. Electrophoretic analysis showed the presence of several components, including a very intense 82 kDa band. This latter component was endowed with intense gelatinolytic activity, as observed in zymography assays. Further analysis revealed that the extract also contained hyaluronidase activity but is devoid of phospholipase A_2_ activity. *In vivo* assays, using mice, showed that the extract was not lethal, but caused significant edema and induced intense infiltration of inflammatory cells to the envenomation site. The extract also induced high specific antibody titers, but no autoantibodies were detected. The data obtained, so far, demonstrate the existence of a mixture of different enzymes in the bristles of *Premolis semirufa* caterpillar, which can act together in the generation and development of the clinical manifestations of the Pararama envenomation.

## Introduction

Moths and butterflies are insects of the Lepidoptera order, of which the young stage is called larva. The larval form of some families of moths containing urticating hairs is known as caterpillar.

Although caterpillar venoms have not been analyzed as much as the venoms from snakes, spiders and scorpions, there are many reports on the characterization of bristles extracts from a variety of species. Coagulation disorders have been reported after contact with the Saturniidae caterpillars from *Lonomia* genus. Since 1989, accidents involving *Lonomia obliqua* species were reported in South of Brazil, Argentine, Paraguay and Uruguay [1], [2]. The physical contact with this caterpillar induces a toxic secretion from bristle, which promotes local and systemic symptoms in the victim between 6 to 72 hours after contact, such as burning sensation, intense hematuria, disseminated intravascular coagulation-like reactions (severe depletion of the coagulation factors) and secondary fibrinolysis [2]. Serious clinical complications, such as acute renal failure and intracranial hemorrhage may also occur [1], [3].

The Brazilian caterpillar of *Premolis semirufa* usually called as *Pararama*, belongs to the Arctiida*e* family. The genus *Premolis* contains four species: *P. semirufa*, recorded in the Amazon region in Brazil, French Guiana, Ecuador, Peru and Panama; *P. excavata* found in Panama; *P. rhyssa* in Peru and *P. amaryllis* in French Guiana.

**Figure 1:**
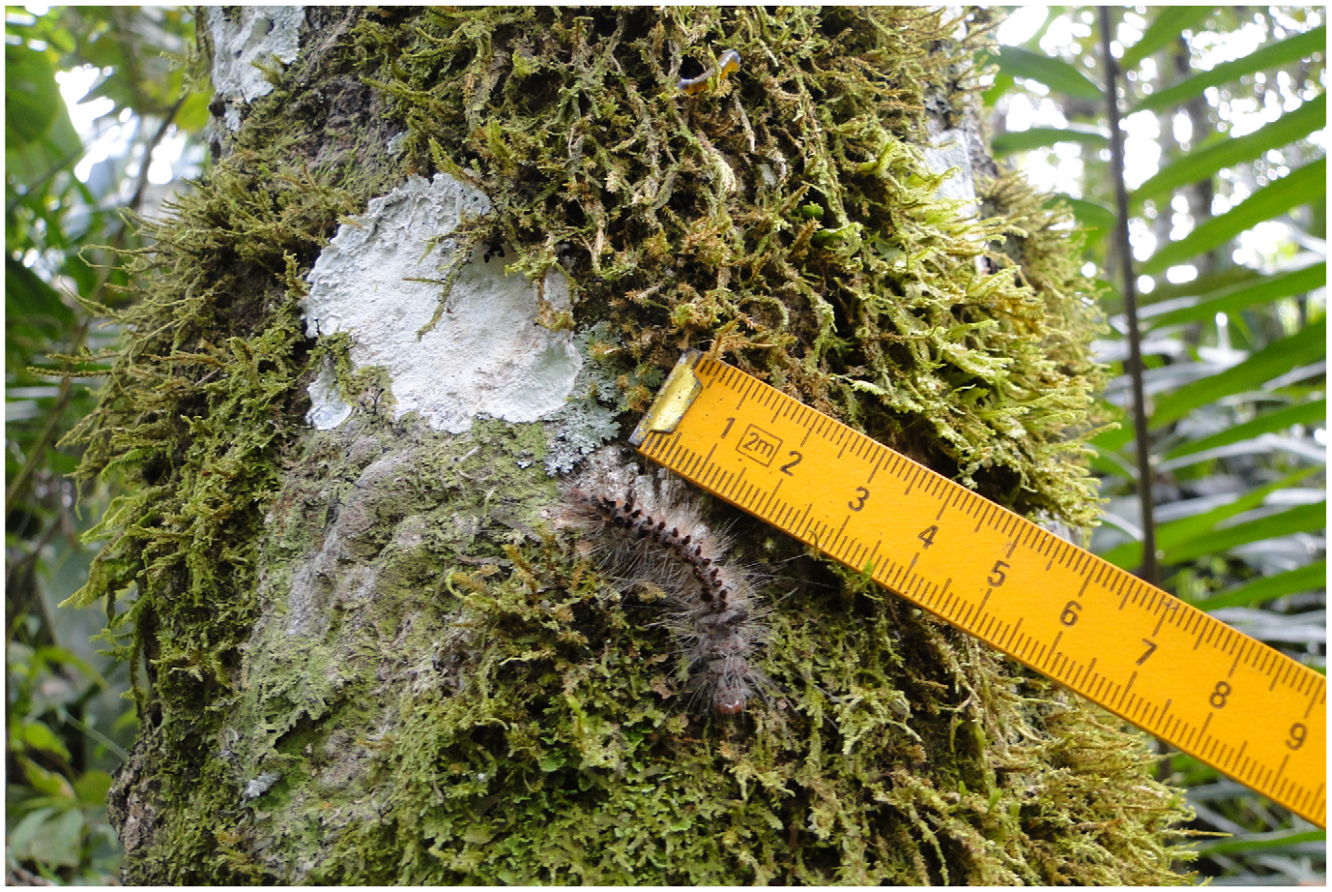
*Premolis semirufa* caterpillar. Pararama in the trunk of *Hevea brasiliensis* in São Francisco do Pará, Pará, Brazil. Photo by Rosana de Fátima Shoji.

*Premolis semirufa* feeds of *Hevea brasiliensis*, the rubber tree found in the Amazon forest (Figure 1). The tappers, when collecting the latex, can stick their fingers in the trunk of the rubber trees to facilitate the harvest and, at that time, may come into contact with the Pararama.

Known as “Pararama associated phalangeal periarthritis” and due to its importance as an occupational disease, predominantly in the rubber tree areas of Pará, Brazil, this caterpillar envenomation was inserted into the “Manual of diagnosis and treatment of envenomations”, released by the Brazilian Ministry of Health in 1992 [4]. The contact with the bristles, in most cases, causes instantly an intense itching, followed by symptoms of the acute inflammation such as pain, heat and redness, which lasts up to seven days, after the first accident [5]–[11]. Chronic symptoms, which frequently occur in individuals after multiple accidents, are characterized by synovial membrane thickening, with joint deformities and chronic synovitis (mono or oligoarticular), symptoms similar as those found in rheumatoid arthritis.

So far, there is no effective treatment for the accidents with Pararama, since neither the toxic components of the bristles of the caterpillar nor the mode of action of the venom are known. However, systemic corticosteroids treatment has been used, in the belief that this would prevent the onset or attenuate the chronic disease [7], [12], [13]. In case of infection, due to the scratching and unhygienic conditions, the disease may progress to pyogenic arthritis [11].

Despite being a serious problem in occupational medicine and a social problem affecting the Brazilian Amazon region, since the rubber tappers can no longer return to their activities, which are the source of their livelihood, studies on the pathogenesis of Pararama are scarce. Thus, the aim of the present study was to analyze the biological and immunochemical characteristics of *Premolis semirufa* caterpillar's bristles crude extract.

## Materials and Methods

### Chemicals and reagents

Triton X-100, Tween-20, Hepes, bovine serum albumin (BSA), cetyltrimethylammonium bromide (CTAB), ortho-phenylenediamine (OPD), phosphatidylcholine, bromothymol blue, gelatin, Coomassie Brilliant Blue R-250, phenylmethylsulfonyl fluoride (PMSF), 1,10-phenanthroline, hyaluronic acid, anti-mouse IgG horseradish labelled with peroxidase (IgG-HRPO), native salmon sperm DNA and collagen from bovine tracheal cartilage were purchased from Sigma–Aldrich (Missouri, USA). Anti-mouse IgM, -IgG1, -IgG2a HRPO-conjugate and anti-mouse IgG2b, IgG3 biotin-conjugate were purchase from BD Bioscience (California, USA). Anti-mouse IgG labelled with alkaline phosphatase (IgG-AP), 5-bromo-4-chloro-3-indolyl-phosphate (BCIP) and nitroblue tetrazolium (NBT) were from Promega Corp. (Wisconsin, USA). Brij-35 P was purchased from Fluka – BioChemika (Werdenberg, Switzerland). Fluorescence Resonance Energy Transfer (FRET) substrates were synthesized and purified according to Araújo *et al.* [14].

### Caterpillar bristles extract

Caterpillars from *Premolis semirufa* were collected in the city of São Francisco do Pará, Pará, Brazil, and maintained at the Immunochemistry Laboratory, Butantan Institute, SP, Brazil. The bristles extract was prepared after exposing the caterpillars to 4°C for few minutes; the bristles were cut off with scissors at their insertion in the tegument, avoiding any tegument incision and, then, suspended in cold phosphate-buffered saline - PBS (8.1 mM sodium phosphate, 1.5 mM potassium phosphate, 137 mM sodium chloride and 2.7 mM potassium chloride, pH 7.2). This suspension was macerated with the aid of a glass stick, homogenized and centrifuged at 560×*g* for 20 min at 4°C. The supernatant was collected and its protein content was determined by using the BCA Protein Assay Kit (Pierce Biotechnology, MA, USA). Supernatant aliquots were stored at −80°C until use. Venoms from *Micrurus hemprichii* and *Bothrops jararaca* snakes, which were used as positive controls in the assays for determination of PLA_2_ and hyaluronidase activities, respectively, were supplied by Herpetology Laboratory from Butantan Institute, SP, Brazil. The authorization to access the venoms of *Premolis semirufa* caterpillar, *Bothrops jararaca* and *Micrurus hemprichii* snakes were provided by the Brazilian Institute of Environment and Renewable Natural Resources - IBAMA - a Brazilian Ministry of the Environment's enforcement agency (permission no. 01/2009).

### Ethics statement

BALB/c strain male mice aged 2 months and weighing 18–22 g were obtained from Central Animal Breeding from Butantan Institute, SP, Brazil. All the procedures involving animals were in accordance with the ethical principles in animal research adopted by the Brazilian Society of Animal Science and the National Brazilian Legislation no. 11.794/08. Protocols were approved by Institutional Animal Care and Use Committee (protocol approval number 413/07).

### Electrophoresis

The caterpillar bristles extract (10 µg of protein) was solubilized in sample buffer, using non-reducing and reducing conditions, and separated on 12% SDS-PAGE gel [15]. Molecular weight markers were included in all runs. Gels were stained with silver [16].

### Phospholipase A_2_ activity

The Phospholipase A_2_ activity of *Premolis semirufa*'s bristles extract was determined a**s** described by Price III [17], with some modifications. Samples of the extra**c**t (4 µg or 16 µg of protein), 20 µL HCl (positive control), or 20 µL PBS (negative control) were mixed in 96-well microtitre plates. 180 µL of an assay mixture containing 10 mM Triton X-100, 5 mM phosphatidylcholine, 1.5 mM HEPES, 10 mM calcium chloride, 0.9% sodium chloride and 0.03% (wt./vol.) bromothymol blue dye in water, at pH 7.5 and 37°C, were added. The plate was analyzed at λ 620 nm in a spectrophotometer (Multiskan EX, Labsystems, Finland) after 5 min of incubation and the linearity of the reaction was verified by linear regression (MSExcel 2007). All enzymatic assays were performed in duplicate and expressed as specific activity (nmol/min/µg). As positive control for PLA_2_ activity, venom of the snake *Micrurus hemprichii* (4.0 µg) was used.

### Hyaluronidase activity

Hyaluronidase activity was measured as described by Pukrittayakamee [18], with slight modifications. In a microtitre plate, *Premolis semirufa*'s bristles extract (8.0 µg of protein) were mixed with 25 µL of the hyaluronic acid (0.5 mg/mL) and acetate buffer (0.2 M sodium acetate-acetic acid, pH 6.0, containing 0.15 M NaCl), in a final volume of 100 µL, and incubated for 30 min at 37°C. After incubation, 200 µL of CTAB 2.5% in NaOH 2% was added to the samples. The absorbances were measured at λ 405 nm in a spectrophotometer (Multiskan EX, Labsystems, Finland) against a blank containing hyaluronic acid, acetate buffer and 250 µL of CTAB. All assays were performed in duplicate. Results were expressed in units of turbidity reduction (UTR) *per* mg of extract. *Bothrops jararaca* snake venom (8.0 µg) was used as positive control.

### Proteolytic activity

#### Zymography

Samples of the extract (0.5 µg of protein) were incubated, at 37°C for 30 min, in the presence or absence of 10 mM 1,10-phenanthroline or PMSF, metallo- and serineproteases inhibitors, respectively, solubilized in non-reducing sample buffer and separated on 10% SDS-PAGE gels containing 1 mg/mL gelatin. The gels were washed for 30 min at room temperature in 2.5% Triton X-100, and incubated for 12 h at 37°C in zymography buffer (50 mM Tris-HCl, 200 mM sodium chloride, 10 mM calcium chloride, 0.05% Brij-35 P; pH 8.3). Following incubation, the gels were stained with 0.2% Coomassie Brilliant Blue R-250. The gelatinolytic activity was detected as unstained bands and densitometry analysis of zymography gels was performed using the Kodak Molecular Imaging Software.

#### Fluorimetric test

The enzymatic activity of the caterpillar bristles extract was determined using the fluorescence resonance energy transfer (FRET) substrate peptide Abz-FRSSRQ-EDDnp. Samples of the extract (1 µg of protein) were mixed with 5 µM of FRET substrate, in cold phosphate-buffered saline (PBS). The relative inhibition was determined in parallel using in the assays 5 mM PMSF or 5 mM 1,10-phenanthroline, inhibitors of serine- and metalloproteases, respectively. The stock solutions and the work concentration of the synthetic inhibitors used in the characterization of the venoms proteolytic activity were made as described [19].

The reactions were monitored by measuring hydrolysis in a fluorescence spectrophotometer (Victor 3™, Perkin-Elmer, MA, USA) using 96-well microtitre plates (λ_em_ = 420 nm and λ_ex_ = 320 nm) at 37°C, as described by Araújo *et al*. [14]. Control samples were prepared in the presence of an equal volume of ethanol, used in inhibitors stock solutions. All assays were performed in duplicate and the specific proteolytic activity was expressed as units of free fluorescence of cleaved substrate *per* min *per* µg of extract (UF/min/µg).

### Determination of the median lethal dose (LD_50_)

Lethality was assessed by intraperitoneal injection of increasing amounts of bristles extract in 200 µL of PBS into male BALB/c strain of mice. Four animals were used for each dose and the LD_50_ was calculated by probit analysis of death occurring within 72 h after extract injection [20].

### Evaluation of the edema

The possible edematogenic activity of the caterpillar bristles extract was evaluated by BALB/c mice intraplantar injection of 50 µL of sterile PBS containing 10 µg (protein) of the extract into the left hind footpad. As control group, mice received 50 µL of sterile PBS into the left hind footpad. The animals were injected, seven times, at intervals of two weeks. Before extract or PBS inoculations, the thickness of each left footpad (Th_0_) was determined using a caliper measurement (Mitutoyo, Sul American Ltda.). Subsequent readings of the thickness (Th_t_) after extract or PBS injections were carried out at 30, 60, 120 and 180 min, and compared to the initial readings. The edema (E) was calculated as follows: E [%] = [(Th_t_−Th_0_)/Th_0_]×100. Where Th_t_ is the thickness (mm) of the footpad at time “*t*” after the injection of the extract or PBS. Th_0_ is the thickness (mm) of the footpad before the injection of the extract or PBS.

### Histopathological analysis

BALB/c mice, injected as described above, were euthanized 24 h after the 7^th^ extract inoculation, their hind limbs removed and processed for histological analysis. The paws were immersed in 10% neutral buffered formaldehyde solution for 24 h. After decalcification, the tissues were embedded in paraffin, sectioned and stained with hematoxylin and eosin (H&E). The H&E preparations were microscopically observed and examined for the presence of inflammatory cell infiltration. As control, paws injected with an equal volume of PBS, were collected, processed and analyzed as described above. All tissue sections were examined under a light microscope (Leica DM2500; Wetzlar, Germany).

### Anti-bristles extract mouse serum

The antiserum against *Premolis semirufa*'s bristles extract was obtained from mice inoculated with 10 µg (protein) of the extract or PBS, into the left hind footpad, seven times at two weekly intervals, without adjuvant. Bleeding was carried out, by retro-orbital plexus with a Pasteur pipette, 48 h after the injection. The blood was allowed to clot at room temperature for 15 min and then was left at 4°C for 6 h. After centrifugation at 560×*g* for 15 min at 4°C, the serum was collected and immediately frozen at −20°C until use.

### Enzyme linked immunosorbent assay (ELISA)

#### Detection of antibodies against bristles extract components

ELISA plates (Costar®, Corning Inc., USA) were coated with 100 µL of the caterpillar bristles extract (10 µg protein/mL; overnight at 4°C). Plates were blocked with 5% BSA in PBS and incubated with dilutions of the anti-caterpillar bristles extract or normal sera obtained from BALB/c mice. After 1 h of incubation at 37°C, plates were washed with PBS/0.05% Tween 20 and incubated with the specific anti-mouse IgG, -IgM, -IgG1 or -IgG2a HRPO-conjugate or with anti-mouse IgG2b, -IgG3 biotin-conjugate for 1 h at 37°C. For biotin conjugated antibodies, an additional step of incubation with HRP-conjugated streptavidin for 30 min at room temperature was carried out. Plates were washed and the reactions developed with OPD substrate, according to the manufacturers conditions (Sigma). The absorbances were recorded in a spectrophotometer (Multiskan EX, Labsystems, Finland) at λ 492 nm. The titer was established as the highest antiserum dilution, which produced an absorbance twice greater than that determined for the normal serum, and expressed as log_2_.

#### For the detection of anti-DNA or anti-Collagen type II antibodies

The presence of anti-DNA or anti-collagen type II IgM and IgG antibodies in sera obtained from mice inoculated or not with the caterpillar bristles extract were also determined by ELISA. Briefly, Immulon 2-HB 96-well flat bottom microtiter plate (Thermo Scientific, NY, USA) were coated with native salmon sperm DNA (1 µg/well), diluted in 50 µL of 10 mM TRIS-HCl, 1 mM EDTA, pH 7.5 or with collagen from bovine tracheal cartilage (3 µg/well), diluted in 100 µL of PBS, and incubated overnight at 4°C. Plates were blocked with 5% BSA in PBS at 37°C for 2 h and dilutions of the sera were added and incubated for 2 h at at room temperature. Sera from [NZBxNZW]F_1_ mice with systemic lupus erythematosus autoimmune disease were obtained as described [21] and used as positive control for the presence of autoantibodies. Plates were washed with PBS/0.05% Tween 20 and incubated with the specific anti-IgG or anti-IgM HRPO-conjugates for 1 h at room temperature. Plates were washed and the reactions developed with OPD substrate, according to the manufacturers conditions (Sigma). The absorbances were recorded in a spectrophotometer (Multiskan EX, Labsystems, Finland) at λ 492 nm.

### Statistical analysis

Statistical analysis was performed by Students't*-test* using GraphPad Prism software. Differences were considered statistically significant when *P* values were *P*<0.05, *P*<0.01 and *P*<0.0001.

## Results

### Eletrophoretic characterization of the *Premolis semirufa*'s bristles extract

**Figure 2:**
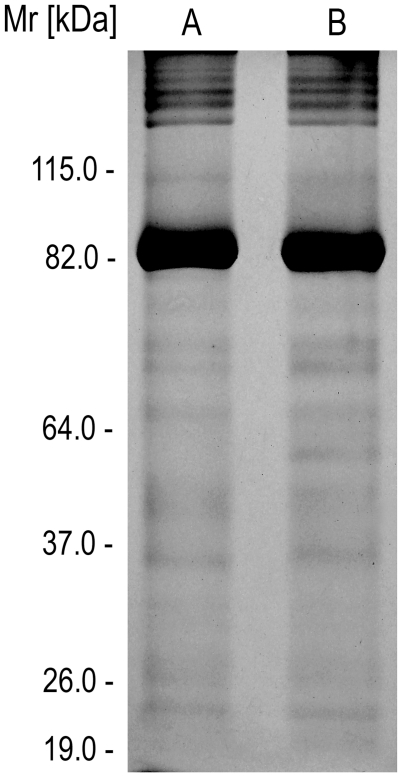
SDS-polyacrylamide gel electrophoresis analysis of *Premolis semirufa*'s bristles extract. Samples (10 µg of protein) of the extract were analyzed by SDS-PAGE gel (12%) under non-reducing [A] and reducing conditions [B] and silver staining.

Extract samples collected from Pararama bristles were prepared in PBS and analyzed for protein composition using SDS-PAGE, under reducing and non-reducing conditions. Figure 2 shows that the electrophoretic profiles of the extract, analyzed under both conditions, were similar, showing components with Mrs between 20 and 200 kDa and the presence of an intense band with Mr around 82 kDa.

### Toxic activities of the *Premolis semirufa*'s bristles extract

In order to assess *Premolis semirufa*'s bristles extract toxicity, the extract was tested using a variety of functional biochemical assays, to identify if it contained activities frequently found in animal venoms. The lethal toxicity of the bristles extract was determined in groups of BALB/c mice, after intraperitoneal injection of increasing protein concentrations of the extracts (1.2 mg/kg, 2.3 mg/kg and 6.8 mg/kg) and no death was observed after 72 hours of the inoculation (data not shown). Moreover, in this condition, no manifestation of discomfort was observed in any of the envenomated animals.

**Figure 3:**
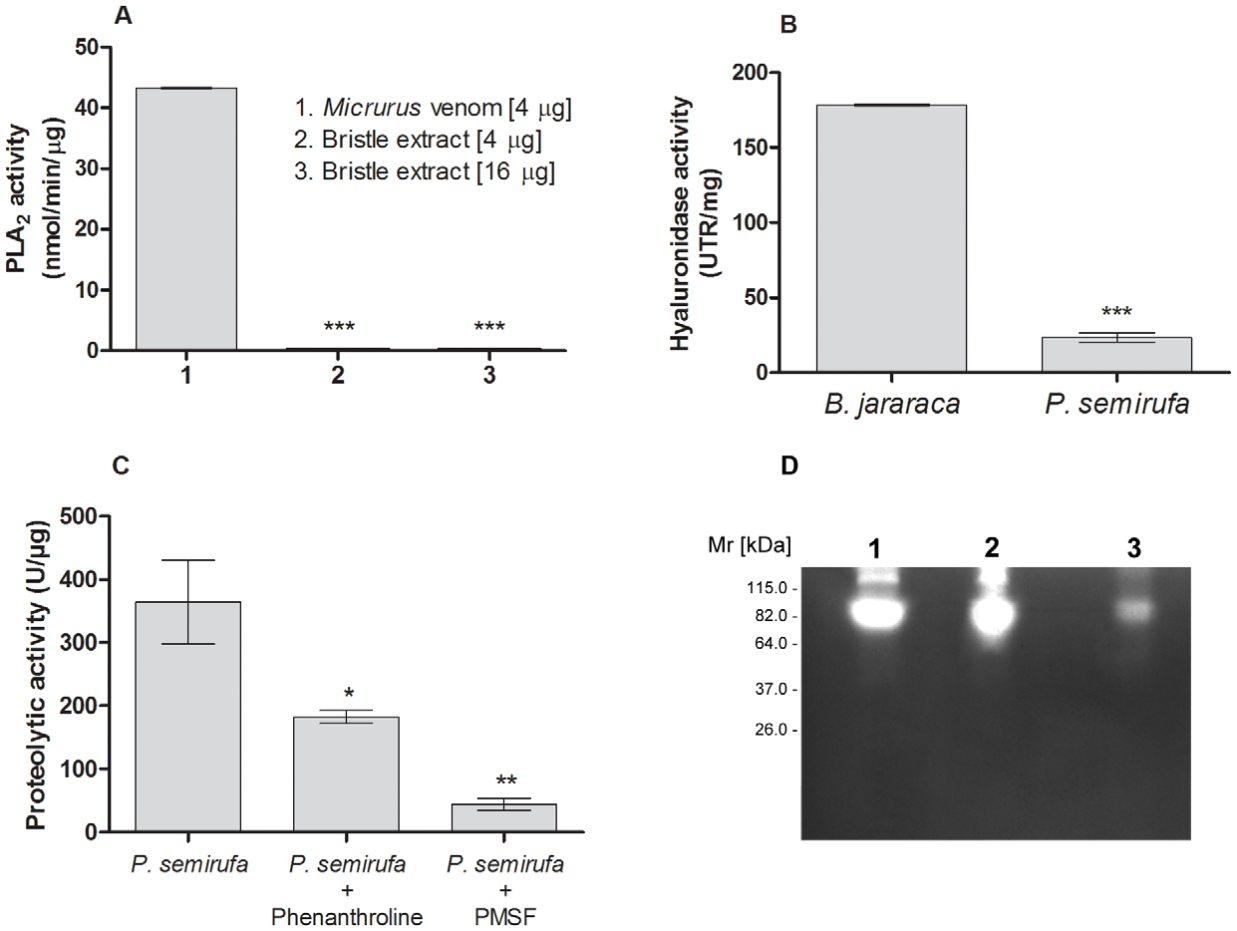
Toxic activities of the *Premolis semirufa*'s bristles extract. [A] PLA_2_ activity: samples of the total extract proteins (4 µg or 16 µg) were incubated, for 5 min at 37°C, with 5 mM phosphatidylcholine. As positive control for PLA_2_ activity, total venom proteins of the snake *Micrurus hemprichii* (4.0 µg) was used. Results are representative for three separate experiments and expressed as nanomoles acid *per* minute *per* µg of venom. *** *P*<0.0001. [B] Hyaluronidase activity: samples of the extract proteins (8.0 µg) were incubated with the hyaluronic acid (0.5 mg/mL) at 37°C for 30 min. As positive control, venom proteins of *Bothrops jararaca* (8.0 µg) was used. The absorbance was measured in a spectrophotometer at λ 405 nm and the hyaluronidase activity expressed as reduction percentage of the turbidity (UTR). Results are representative for three separate experiments and expressed as specific activity (UTR/mg) ± SD. *** *P*<0.0001. [C] Proteolytic activity determined by using Abz-FRSSRQ-EDDnp as substrate: samples of the bristles extract proteins (1 µg), pre-incubated or not for 20 min with 5 mM PMSF or 5 mM 1,10-phenanthroline, were incubated with the FRET substrate (5.0 µM). All enzymatic assays were performed in duplicate and the results expressed as specific activity (U/mg) ± SD. *P<0.05 and ** *P*<0.01: significant differences between the mean values obtained with the extract and the mean values obtained with the inhibitors. [D] Proteolytic activity analyzed by zymography: samples (0.5 µg of protein) of the extract were incubated in the absence (line 1) or presence of 10 mM 1,10-phenanthroline (line 2) or PMSF (line 3), inhibitors of metallo - and serine - proteases, respectively, submitted to electrophoresis and subsequently incubated, at 37°C for 12 h, in 50 mM Tris-HCl, pH 8.3. Following incubation, the gel was stained with 0.2% Coomassie Brilliant Blue R-250 and the gelatinolytic activity was detected as unstained bands on a dark background.

The phospholipase A_2_ (PLA_2_) activity of *P. semirufa* caterpillar bristles extract was assessed by a colorimetric method after incubating samples of 4 or 16 µg of the extract with phosphatidylcholine, the substrate of the PLA_2_. Figure 3A shows that the venom of the caterpillar showed no PLA_2_ activity, while the *Micrurus* snake venom (4 µg), used as positive control, presented a high lipase activity on phosphatidylcholine.

The hyaluronidase activity of the caterpillar bristles was measured incubating samples of the extract (8 µg of protein) with hyaluronic acid, the substrate of the reaction. As positive control of the reaction, it was used the venom from *Bothrops jararaca* snake. Figure 3B shows that the bristles extract present significant hyaluronidase activity.

The proteolytic activity of the bristles extract (1 µg) was tested using the fluorescence resonance energy transfer (FRET) peptide Abz-FRSSRQ-EDDnp as substrate. Figure 3C shows that the extract efficiently hydrolyzed the FRET peptide, and that this activity was strongly inhibited by the serine protease inhibitor PMSF (88%) and partially by the metalloprotease inhibitor phenanthroline (50%).

Figure 3D shows that a 82 kDa component, the Mr corresponding the intense protein band observed after silver staining (Figure 2), has a high gelatinolytic activity, as measured by zymography (Fig. 3 - line 1). This activity was significantly inhibited by PMSF (Fig. 3 - line 3), a serineprotease inhibitor, and poorly blocked by phenanthroline (Fig. 3 - line 2), a metalloprotease inhibitor.

### Edema inducing activity

**Figure 4:**
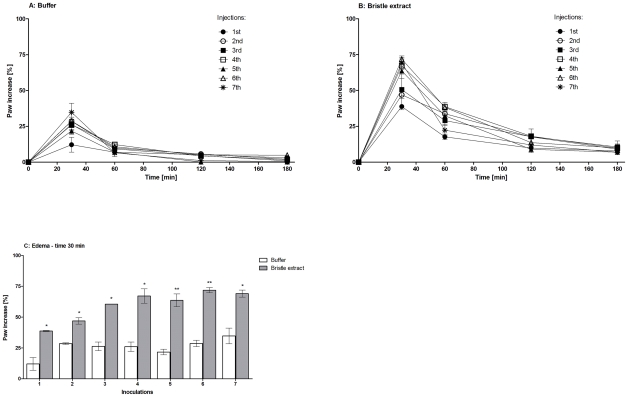
Edematogenic action of *Premolis semirufa's* bristles extract. Edema was induced by intraplantar administration of 0.05 ml of sterile PBS [A] or samples of 10 µg of protein of the caterpillar bristles extract [B] into the left hind footpad. Paw edema was determined, by measuring paw thickness using a caliper at 0, 30, 60, 120 and 180 min after administration of extract or buffer. [C] Comparison of the edematogenic responses, after 30 min of PBS or extract injections, during the seven inoculations. Results were calculated by the formula: E(%) = [(Th_t_−Th_0_)/Th_0_]×100; where Th_t_ is the thickness (mm) of the rear left footpad at “t” time after the injection of the extract or PBS, Th0 is the thickness (mm) of the rear left footpad before the injection of the extract or PBS. *P<0.05 and ** P<0.01: significant differences between the mean values of buffer group and the mean values of *P. semirufa* group.

BALB/c mice were injected seven times, at intervals of two weeks, with 10 µg of the extract proteins into the foot pad of the left hind leg. Controls animals were injected with PBS. The intraplantar injection of *Premolis semirufa* caterpillar bristles extract, caused discomfort to the animals (pain) and a significant increase in the paw volume, as compared to that induced by injection of the vehicle, *i.e*., PBS (Figure 4A and B). The edema induced by both, extract and PBS, was detected as early as 5 min post-injection and peaked at 30 min. The increase in paw volume was observed until 300 min after injection of the extract, while the increase induced by PBS was resolved 120 min after injection. The comparison of the edematogenic responses, along the seven inoculations of the extract or PBS, determined at the peak of the reaction, *i.e.*, at 30 min is shown in Figure 4C. The edematogenic responses were significant and successively more intense after the inoculations of the extract compared to PBS and reached a maximum after the 4^th^ injection.

### Histopathological analysis

**Figure 5:**
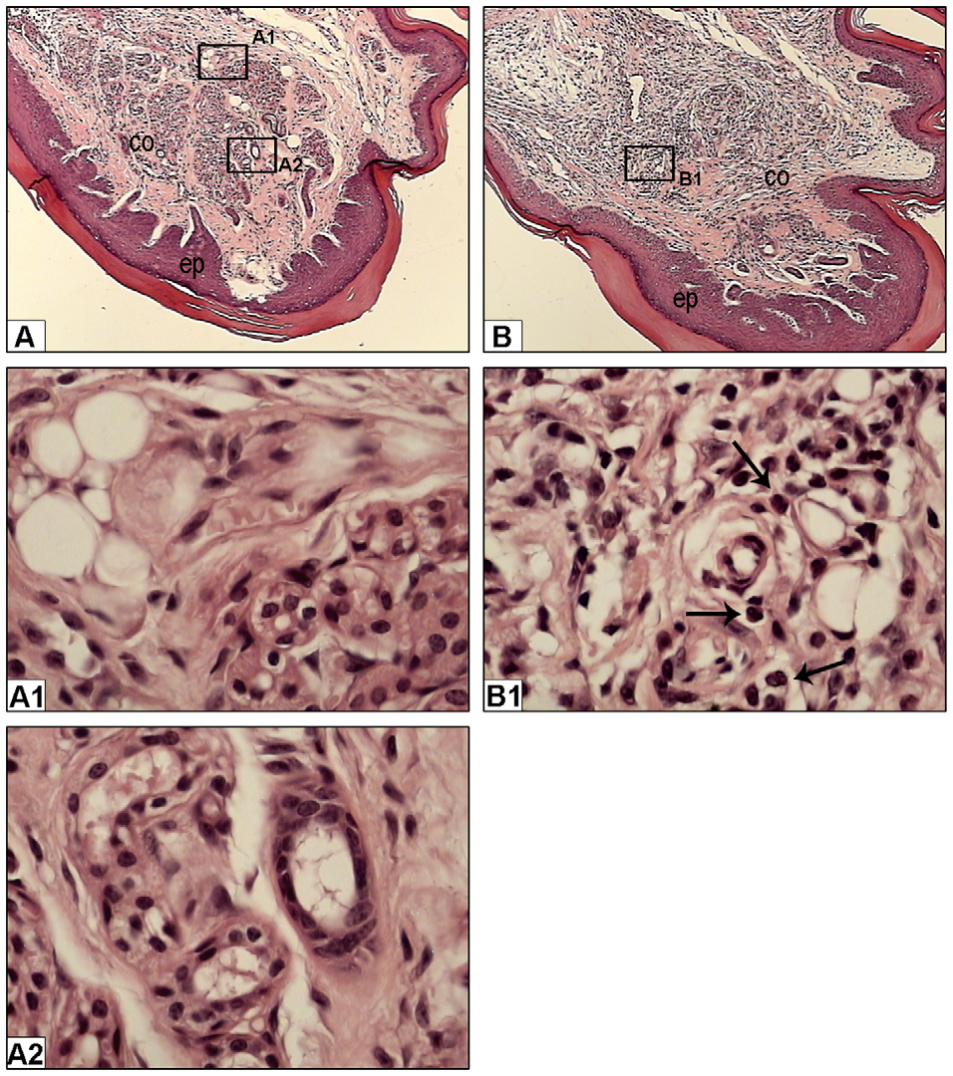
Analysis of the inflammatory process induced by *Premolis semirufa's* bristles extract. Mice were injected with samples of 10 µg of protein of bristles extract or with PBS, seven times, at intervals of two weeks and 24 h after the last injection, the whole paws were harvested from euthanized animals, sectioned and stained with hematoxylin and eosin (H&E). Panels correspond to paws sections from mouse injected with PBS [A, A1, A2] and *Premolis semirufa's* bristles extract [B, B1]. Epithelial (ep), connective (co), fat tissue [Panel A: rectangle A1] and sweat glands [Panel A: rectangle A2]. Note that the fat tissue and sweat glands were replaced by connective tissue in the bristles extract injected paws [panels B and B1]. In controls, all structures remained preserved and there was no inflammatory process in the paws sections from mouse injected with PBS [Panels: A, A1 (zoom in the rectangle A1 from panel A) and A2 (zoom in the rectangle A2 from panel A)]. In bristles extract injected paws a marked inflammatory cell infiltration, consisting of neutrophils was observed (Panel B1: thin arrows), while the amount of fat tissue and sweat glands was reduced [Panels: B and B1 (zoom in the rectangle B1 from panel B)]. Panels A and B: original magnification ×100; Panels A1, A2 and B1: original magnification ×1000.

BALB/c mice, injected as described above, were euthanized 24 h after the 7^th^ extract or PBS inoculations, their hind limbs removed and processed for histological analysis. Figure 5B shows that the bristles extract injection resulted in the establishment of a pronounced inflammatory reaction, characterized by the presence of mixed inflammatory cellular infiltrate distributed throughout the tissue. Furthermore, the connective tissue was increased, partially occupying areas where, in normal tissues, structures such as sweat glands and fat tissue were found (Figures 5A and C), and initiating a fibrotic process (Figure 5D).

### Immunogenicity of the Pararama bristles extract antiserum

**Figure 6:**
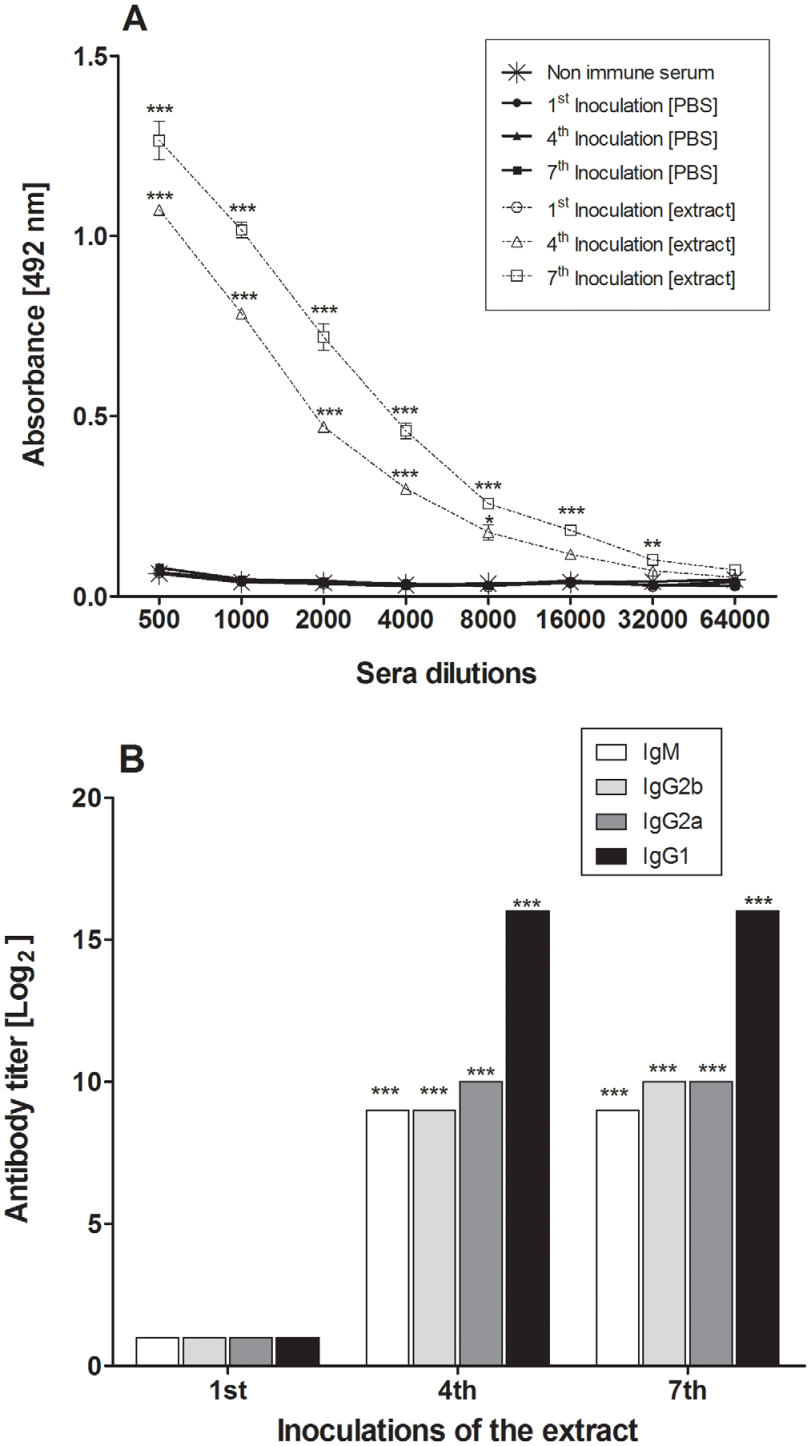
Immunogenicity of Pararama bristles extract. [A] ELISA plates were coated with 1.0 µg of extract proteins/well and incubated with different dilutions of the sera obtained from BALB/c mice inoculated with the extract or PBS, one, four or seven times, followed by anti-mouse IgG-HRPO-conjugated (1∶30,000). The results were expressed as the mean of absorbance value ± SD. ** *P*<0.01; ***P<0.0001: significant differences between the sera obtained after extract or PBS inoculations and pre-immune serum. [B] Determination of IgG1, IgG2a, IgG2b and IgM antibody titers of sera collected from mice inoculated with extract, by ELISA. The titers were established as the highest antiserum dilution which produces an absorbance twice greater than that determined for the normal serum and expressed as log_2_. ***P<0.0001: significant differences between the titers obtained for IgG1 and the other antibodies.

The immunogenicity of *Premolis semirufa* caterpillar bristles extract was assessed by ELISA, using sera obtained from BALB/c mice subcutaneously inoculated with 10 µg of the extract proteins or PBS. Figure 6A shows that the repeated inoculations of the extract, but not of PBS, induced a high IgG antibody response. In addition, analysis of antibody classes and subclasses revealed that the sera obtained from animal injected with the extract presented higher IgG1, IgG2a, IgG2b and IgM titers as compared to the sera collected from PBS injected mice. IgG1 sera titers, determined for envenomated animals, were higher than the others antibodies isoptype/sucblcasses and no IgG3 antibodies could be detected in these samples (Figure 6B).

### Detection of the anti-DNA and anti-Collagen type II autoantibodies

The presence of anti-DNA or anti-Collagen type II IgM and IgG autoantibodies in sera from BALB/c mice inoculated with extract or PBS, was evaluated by ELISA. Anti-DNA or anti-Collagen type II IgG antibodies were not detected in sera from bristles extract inoculated animals, while high titers of these antibodies were detected in the serum of mice with systemic lupus erythematosus (SLE) autoimmune disease. Anti-DNA and anti-Collagen type II IgM antibodies were not detected in bristles extract injected or SLE mice (data not shown).

## Discussion

We have investigated activities of the bristles extract of Pararama, a caterpillar responsible for the occupational disease ‘Pararama associated phalangeal periarthritis’. Until now nothing was know about the composition of its venom.

In this paper we present, for the first time, some of the biochemical and biological properties of *Premolis semirufa*'s bristles extract. Electrophoretic analysis of the extract showed that it contained a great diversity of proteins, with Mr ranging from 20 to 200 kDa, with a major protein band of 82 kDa, contributing to over 90% of the protein content. No significant difference in the protein profile was observed in the extracts submitted to reducing or non-reducing conditions.

We subjected the venom to a variety of functional biochemical assays, to identify if it contained activities frequently found in animal venoms. Hyaluronidase activity is present in many animals venoms and its activity potentiates the toxicity of the venom, promoting loss of extracellular matrix integrity of soft connective tissues, surrounding the blood vessels, increasing the systemic influx of toxins and, thus, facilitating the dispersion of the toxic components [22]. *Premolis semirufa*'s bristles extract showed significant hyaluronidase activity, suggesting that this enzyme may participate in the genesis of the joint immobility, since hyaluronic acid is an abundant component of the intercellular matrix of the skin, cartilage and synovial fluid, playing an important role as stabilizer and lubricant of the joints [23]. The hyaluronic acid degradation may explain, in part, the changes in the joint and loss of the cartilage and bone structure, seen in the pararama induced disease.

Zymography analysis showed that the 82 kDa component found in the caterpillar bristles extract possesses gelatinolytic activity. Gelatinases are capable of degrading types IV, V, VII and XI collagens, present in bone and articular cartilage, and may regulate their remodeling [24]. Using specific inhibitors for metallo- and serine- proteases we identified the gelatinase as a serine protease. The bristles extract also demonstrated high proteolytic activity towards the FRET peptide Abz-FRSSRQ-EDDnp. The use of PMSF showed that serineproteases were largely responsible for this while using the metalloproteases inhibitor phenanthroline demonstrated that metalloproteases were involved as well. Venom serineproteinases have a highly diverse pharmacological potential, including actions on proteins of the coagulation cascade, activation of factor V, activation of protein C, fibrinogenolysis, activation of the plasminogen and induction of platelet aggregation [25]. Thus, it is possible to propose that serineproteases, with gelatinolytic activity and other proteolytic activities, may be involved in the process of cartilage and joint degradation produced by contact with the bristles of Pararama [26]. Metalloproteinases, abundant molecules in snake venoms, are responsible for the development of local tissue injury and the occurrence of bleeding [27], being able to degrade important components of the matrix, such as laminin and type IV collagen [28]. Thus, the presence of different classes of proteases in the Pararama bristles extract may contribute to the tissue injury seen in the caterpillar human accidents.

In many animal venoms, Phospholipase A_2_ (PLA_2_) is important for digestion and immobilization of the prey, as well as responsible for some pathologies observed in humans stung/bitten by bees, wasps, spiders and snakes [29]–[31]. Phospholipase A_2_ activity has also been described in crude bristles extract of the *Euproctis* (Lymantriidae) caterpillar [32] and more recently in bristles crude extract of *Lonomia obliqua* (Saturniidae) [33]. However, *Premolis semirufa*'s bristles extract did not show phospholipase A_2_ activity.

The present study also aimed to evaluate the toxicity of the bristles extract using a murine model and, under the experimental conditions used, no discomfort or death was observed. On the other hand, the intraplantar injection of the bristle extract, as used in histopatological/edema studies, caused a strong discomfort to the animals, suggesting that they were feeling pain. Further studies will be conducted in order to analyze the possible hyperalgesic properties of the pararama venom. Toxic venom proteins serve in a number of adaptive functions such as immobilizing, paralyzing, killing, liquefying prey and deterring competitors. Other proteins may act synergistically by enhancing the activity or spreading of toxins. In contrast to animals such as snakes and scorpions, which use venoms to immobilize prey and to facilitate its digestion, caterpillars feed on leaves; their venoms are used solely for defense [34] and, therefore, has not to be necessarily lethal.

The first intraplantar injection of *Premolis semirufa*'s bristles extract produced a swelling which was detected after 5 min, peaked at 30 min and disappeared within 300 min after injection, while the response to PBS disappeared within 120 min. The prolonged and increased induction of the edema upon 1^st^ exposure was likely to be mediated by the action of the venom, being pro-inflammatory itself or by inducing inflammatory mediators, locally released or synthesized in the course of the envenomation, all of which would increase the permeability of the microvessels. The response induced 30 min after extract injection, gradually increased over the inoculations and reached a maximum after 4 injections. This response was significantly higher than the response to PBS. In addition, the multiple extract injections in mice footpads induced a pronounced inflammatory reaction, characterized by the presence of mixed inflammatory infiltrate, with increase of the conjunctive tissue and beginning of the fibrosis process. In a previous study using a rat model, Costa and collaborators [8] have shown that inflammation was induced by the injection of saline extract of pararama bristles, with the presence of a large number of inflammatory cells around the site of injury.

Investigation on the *P. semirufa*'s bristles extract immunogenicity revealed that the repeated inoculations of the extract, in the absence of adjuvants, induced a strong immune response, with high antibody titers. Moreover, data showed that the sera obtained from animal injected with the extract presented higher IgG1 titers than other IgG subclasses, indicating the predominance of a Th2 immune response, since this particular antibody subclass is mainly induced by the presence of Th2 cytokines such as IL-4, IL-5, IL-10 and IL-13 [35].

The disease caused by the contact with the *Premolis semirufa*'s bristles shares many features with those found in patients with rheumatoid arthritis (RA), a systemic and chronic illness, characterized by severe synovial inflammation and cartilage and/or bone destruction [36]. Autoantibodies, such as anti-collagen type II and anti-DNA, are found in the vast majority of patients [37]. Analysis of the presence of anti-DNA or anti-collagen type II antibodies revealed that these autoantibodies were not present in the sera obtained from mice inoculated with the *Premolis semirufa*'s bristles extract.

Together, these data show the existence, in the *Premolis semirufa*'s bristles extract, of a mixture of different enzymes that may be acting together in the generation and development of clinical disease manifestations. Moreover, this study demonstrates the production of high antibody titers in mice inoculated with the extract, which may also contribute to genesis of inflammatory reactions observed in the envenomation. The absence of autoantibodies indicate that the molecular mechanisms causing disease after multiple contact with the *Premolis semirufa*'s bristles differ from that observed in chronic synovitis, such as the rheumatoid arthritis. The bristles toxic action, high antibody response with the formation of immune complexes and complement activation may also play a role in the establishment of the disease. These aspects will be further investigated in future studies.
